# Supplementary material for: Redox Imbalance and Oxidative DNA Damage During Isoniazid Treatment of HIV-Associated Tuberculosis: A Clinical and Translational Pharmacokinetic Study
Source: Front Pharmacol. 2020 Jul 29;11:1103. doi: 10.3389/fphar.2020.01103 (PMC7406860; doi:10.3389/fphar.2020.01103)
Supplement: Supplementary Figure 1 — Physiologically-based pharmacokinetic model of isoniazid and metabolites. [file DataSheet_1.docx]

**Supplementary Figure 1.** Physiologically-based pharmacokinetic model of isoniazid and metabolites.

**Supplementary Table 1.** Parameters used in the physiologically-based pharmacokinetic model of isoniazid and metabolites.

| **Tissue Compartment** | **Volume (L)** | **Simulated K_p_** | | | |
| --- | --- | --- | --- | --- | --- |
|  |  | **Isoniazid** | **Acetylisoniazid** | **Hydrazine** | **Acetylhydrazine** |
| Lung | 1.14 | 0.72 | 0.70 | 14.31 | 0.79 |
| Arterial supply | 2.11 | 0 | 0 | 0.00 | 0.00 |
| Venous return | 4.22 | 0 | 0 | 0.00 | 0.00 |
| Adipose | 21.00 | 0.17 | 0.17 | 1.55 | 0.18 |
| Muscle | 23.96 | 0.66 | 0.64 | 6.48 | 0.73 |
| Liver | 1.53 | 0.67 | 0.65 | 16.54 | 0.74 |
| Spleen | 0.17 | 0.70 | 0.67 | 11.96 | 0.77 |
| Heart | 0.33 | 0.71 | 0.69 | 8.71 | 0.78 |
| Brain | 1.49 | 0.70 | 0.69 | 2.68 | 0.77 |
| Kidney | 0.35 | 0.70 | 0.67 | 18.06 | 0.76 |
| Skin | 2.73 | 0.66 | 0.65 | 5.43 | 0.71 |
| Reproductive organs | 0.05 | 0.70 | 0.68 | 18.06 | 0.77 |
| Red marrow | 1.07 | 0.41 | 0.41 | 3.00 | 0.43 |
| Yellow marrow | 2.97 | 0.17 | 0.17 | 1.55 | 0.18 |
| Rest of body | 2.50 | 0.70 | 0.68 | 11.96 | 0.77 |

**Supplementary Table 2.** Properties of HPLC-MS/MS plasma assays for isoniazid and metabolites.

| **Analyte** | **Lower limit of quantification (LLOQ, ng/mL)** | **Limit of detection (LOD, ng/mL)** | **Inter-day variability**  **(% Bias)** | **Intra-day variability**  **(% Bias)** |
| --- | --- | --- | --- | --- |
| Isoniazid | 1 | 0.8 | 3.0 | -1.6 |
| Acetylisoniazid | 10 | 6.4 | 2.0 | 2.1 |
| Hydrazine | 2 | 0.4 | -3.0 | -8.5 |
| Acetylhydrazine | 10 | 2.0 | -3.5 | -3.4 |

**Supplementary Table 3.** GSTA2 haplotype designations based on SNPs, according to naming schema proposed by Tetlow *et al*^23^. The rs2234951 and rs1803682 variants were not detected in the patient cohort; rs6577 and rs2180314 were in complete linkage disequilibrium.

| ***GSTA2* haplotype** | **rs2234951 (P110S)** | **rs2180314 (S112T)** | **rs1803682 (K196N)** | **rs6577 (A210C)** |
| --- | --- | --- | --- | --- |
| GSTA2*A | **-** | **-** | **-** | **-** |
| GSTA2*B | **-** | **-** | **-** | **+** |
| GSTA2*C | **-** | **+** | **-** | **-** |
| GSTA2*D | **-** | **-** | **+** | **-** |
| GSTA2*E | **+** | **-** | **-** | **-** |
